# Exploring the Rhizospheric Microbial Communities under Long-Term Precipitation Regime in Norway Spruce Seed Orchard

**DOI:** 10.3390/ijms25179658

**Published:** 2024-09-06

**Authors:** Dagmar Zádrapová, Amrita Chakraborty, Petr Žáček, Jiří Korecký, Anirban Bhar, Amit Roy

**Affiliations:** 1 Faculty of Forestry and Wood Sciences, Czech University of Life Sciences, Kamýcká 129, Suchdol, 165 21 Prague, Czech Republic; 2 Faculty of Science, Charles University in Prague, BIOCEV, Průmyslová 595, Vestec, 252 42 Prague, Czech Republic; 3 Molecular Plant and Microbiology Laboratory (MPML), Post Graduate Department of Botany, Ramakrishna Mission Vivekananda Centenary College, Rahara, Kolkata 700118, India

**Keywords:** precipitation, rhizosphere, microbial communities, amplicon sequencing, soil metabolites, Norway spruce, seed orchards, network analysis, PICRUSt2, FUNGuild

## Abstract

The rhizosphere is the hotspot for microbial enzyme activities and contributes to carbon cycling. Precipitation is an important component of global climate change that can profoundly alter belowground microbial communities. However, the impact of precipitation on conifer rhizospheric microbial populations has not been investigated in detail. In the present study, using high-throughput amplicon sequencing, we investigated the impact of precipitation on the rhizospheric soil microbial communities in two Norway Spruce clonal seed orchards, Lipová Lhota (L-site) and Prenet (P-site). P-site has received nearly double the precipitation than L-site for the last three decades. P-site documented higher soil water content with a significantly higher abundance of Aluminium (Al), Iron (Fe), Phosphorous (P), and Sulphur (S) than L-site. Rhizospheric soil metabolite profiling revealed an increased abundance of acids, carbohydrates, fatty acids, and alcohols in P-site. There was variance in the relative abundance of distinct microbiomes between the sites. A higher abundance of Proteobacteria, Acidobacteriota, Ascomycota, and Mortiellomycota was observed in P-site receiving high precipitation, while Bacteroidota, Actinobacteria, Chloroflexi, Firmicutes, Gemmatimonadota, and Basidiomycota were prevalent in L-site. The higher clustering coefficient of the microbial network in P-site suggested that the microbial community structure is highly interconnected and tends to cluster closely. The current study unveils the impact of precipitation variations on the spruce rhizospheric microbial association and opens new avenues for understanding the impact of global change on conifer rizospheric microbial associations.

## 1. Introduction

Forests cover the largest terrestrial surface on Earth, with an estimated area of ~40 million square kilometers comprising more than 3 trillion trees [1,2]. Forests are an integral component that act as carbon sinks and nutrient cycling [3]. The transformation of organic matter and other processes in soil depends mainly on the soil microbial communities that are pivotal in maintaining the proper functioning of the forest ecosystem [4,5,6]. The rhizosphere is the interface between the living roots and the bulk soil, considered a microbial hotspot. The rhizospheric microenvironment encompasses diverse microbial communities that play a pivotal role in plant growth and health by enhancing nutrient acquisition in plants from the soil, producing growth-promoting plant hormones, protecting against pathogen infection, and conferring tolerance to abiotic stress [7]. Nevertheless, the rhizospheric soil microbial communities contribute to several ecosystem processes, including soil organic matter decomposition, biogeochemical cycling, and carbon sequestration [8]. Hence, any alterations to the rhizospheric microbial communities primarily affect plant growth and ecosystem functioning [9].

Several biotic and abiotic factors, such as host plant species (genotypes and physiological stage), soil physicochemical properties, and climate, shape the rhizosphere microbiome [10,11,12]. Over the years, extensive anthropogenic activities have led to an increase in the temperature of the Earth’s surface to 1.1 °C [13], which has intensified the global hydrological cycle and altered precipitation patterns [14]. Since temperature and water availability are crucial for sustaining life, altering such drivers could significantly impact biodiversity [15]. Global climate change is one of the critical drivers that profoundly impacts soil microbial communities, influencing ecosystem functioning [16]. Alteration in precipitation patterns is a major concern in the context of global climate change. Precipitation change can affect the soil microbiota by altering the soil moisture content, nutrient availability, and plant community [17,18]. Water availability is essential for soil microbial growth and optimal activity [19,20]. Limitations to water availability decrease microbial activity, biomass, and plant growth. Hence, precipitation patterns and global warming changes can alter the microbial community structure and function [21].

Interestingly, rhizosphere microbiota show succession patterns and phylogenetic conservation of rhizospheric competence characteristics, suggesting evolutionary adaptation to host plant species [22]. However, most of the studies are limited to agricultural ecosystems where the in-depth understanding of the stable rhizosphere microbiota is restricted due to the short growth period of the crops [23,24]. On the contrary, conifers establish their rhizospheric microbial associations over prolonged interactions with the soil environment in the forest ecosystems, providing resistance to biotic and abiotic stresses [25]. In addition, root exudates ubiquitous in the rhizosphere enrich beneficial microbial taxa in the proximal soil that promote resistance to pathogen invasion in plants and confer tolerance to biotic and abiotic stresses [26]. Interestingly, such belowground associations are also fine-tuned based on the tree species [27]. Few studies have explored rhizosphere soil microbial communities within forest ecosystems and their responses to climate change [5,28,29,30,31]. Moreover, information on the ecological functions of rhizosphere microorganisms in forest soils under long-term differing precipitation regimes is scarce. Gaining a deeper understanding of the dynamics and mechanisms governing these communities, particularly in response to changes in precipitation regime, is crucial for improving predictions of how climate change will impact the ecological functions of soil microbes.

The present study aims to explore the long-term precipitation change for over three decades on the rhizospheric soil microbial communities from two Norway spruce seed orchards. The two seed orchards are fascinating as they constitute grafted clonal Norway spruce varieties and are not random forest sites. The two forest sites were located at a distance of 25 km (air distance) with an altitude difference of 400 m. The annual precipitation and the temperature monitored over three decades documented different precipitation levels (nearly double) between the two sites with a temperature difference of ~1.6 °C. We hypothesize that variation in the precipitation regime will impact the microbial diversity as well as the soil metabolite profile between the two forest sites. The forest site receiving higher precipitation has high soil moisture content and microbial activity, leading to a higher abundance of soil metabolites. The high soil moisture content will also impact the microbial community structure. In this light, we investigated rhizospheric soil physiology (soil texture, moisture content, selected elements), metabolites, and microbial diversity to apprehend the response to long-term precipitation variation. Our results revealed the impact of precipitation on rhizospheric soil properties, metabolites, and soil microbiota, providing insights into the functioning of the forest soil ecosystem under varied precipitation regimes.

## 2. Results

### 2.1. Soil Texture and Physicochemical Properties

**Figure 1:**
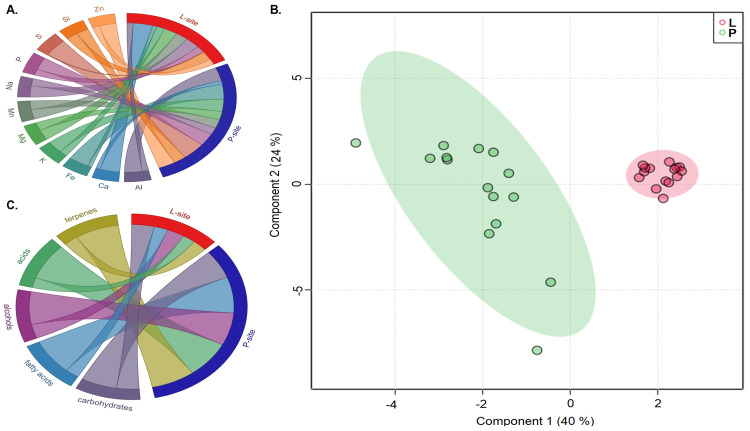
(**A**) Amount of selected elements (in percentage) present in rhizospheric soil samples from Lipová (L-site) and Prenet (P-site) (**B**) Relative abundance of different classes of metabolites (in percentage) present in the soil samples from the two sites. (**C**) Sparse partial least square discriminant analysis (sPLS-DA) plot representing the differences in the metabolite profiles in rhizospheric soil samples from two different sites, Lipová site (L) and Prenet site (P), with fifteen replicates (3 replicates from each clonal tree variety).

The determination of soil texture based on the distribution of soil particle size revealed that the rhizospheric soil from Lipová (L) site (0.03% clay, 23.4% silt, 76.5% sand) and the Prenet (P) site (0.05% clay, 29.4% silt, 70.4% sand) had loamy sand texture. The moisture content in the rhizospheric soil from the P-site (38%) was higher than the L-site (13%), which might be due to the increased precipitation at the P-site. Estimating the amounts (ppm) of different elements (Al, Ca, Fe, K, Mg, Mn, Na, S, P, Si, Zn) present in the soil showed that Aluminium (Al), Iron (Fe), Phosphorous (P) and Sulphur (S) were significantly abundant (*t*-test, *p* < 0.05) in the P-site compared with the L-site. However, there was no significant difference in the amounts of the other elements under study between the sites (Figure 1A, Excel S1).

### 2.2. Metabolite Profiling

The rhizospheric soil metabolite profiling from the two seed orchards was clustered separately into distinct groups, as represented by the component analysis in the Sparse PLS discriminant plot (Figure 1B). The presence of 204 metabolites primarily comprising fatty acids, alcohols, carbohydrates, and acids was documented in this study, of which the heatmap illustrated the top 50 significantly abundant metabolites (*t*-test, *p* < 0.05) (Figure S1, Excel S1). The chordial plot illustrates the relative amount of major metabolite classes in the rhizospheric soil samples from two sites (Figure 1C). The rhizospheric soil documented a high abundance of carbohydrates (L-site 62.7%; P-site 55%), alcohols (L-site 17%; P-site 22%), fatty acids (L-site 2.5%; P-site 4.5%), acids (L-site 0.85%; P-site 1.7%), and terpenes (L-site 2.7%; P-site 4.3%). The metabolite classes acids, fatty acids, and terpenes were significantly abundant in the P-site (*t*-test, *p* < 0.05). It is worth noting that the presence of soil metabolites is attributed to the soil organic matter, plant exudates, and microbial metabolites; however, it is difficult to differentiate the contribution of individual driving factors to the soil metabolite profiles [32].

### 2.3. Rhizospheric Soil Microbial Community Structure

#### 2.3.1. Sequencing Results

The Illumina paired-end amplicon sequencing targeting the bacterial 16S rRNA gene and the fungal ITS2 region yielded 12,945,042 and 8,730,722 reads representing the bacterial and the fungal diversity in the soil samples from the two seed orchards, respectively. The clean reads (9,630,977 clean bacterial reads; 8,253,265 clean fungal reads) were further processed using bioinformatic data analysis pipelines in QIIME2 (Excel S2).

#### 2.3.2. Microbial Communities in Soil

**Figure 2:**
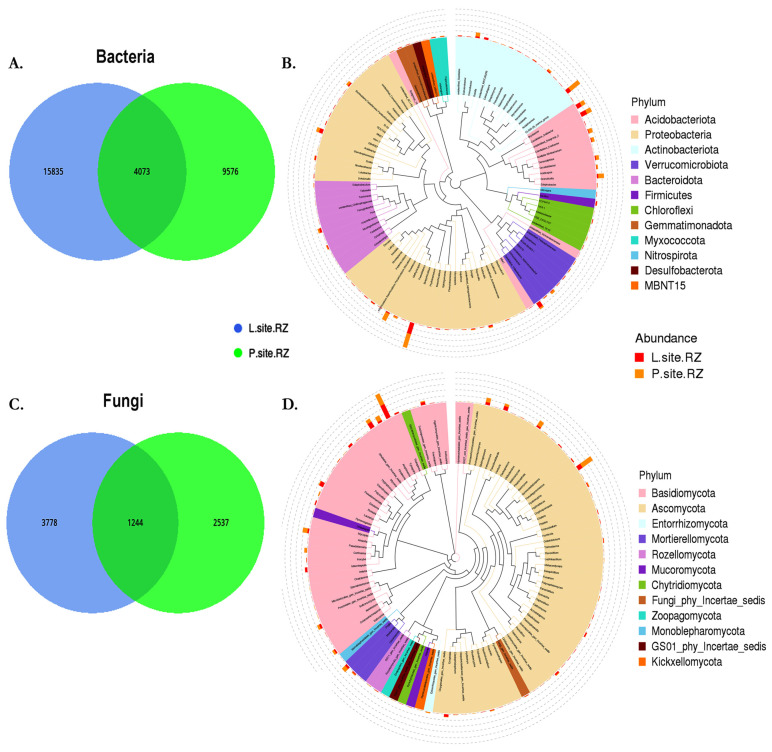
(**A**) Venn diagram showing the distribution of unique and common bacterial ASVs shared between two different rhizospheric soil samples (Lipová and Prenet soil). (**B**) The evolutionary tree representing the top 100 bacterial genera in soil samples (**C**) Venn diagram showing the distribution of unique and common fungal ASVs shared between the two different soils. (**D**) The evolutionary tree representing the top fungal genera present in rhizospheric soils from two sites (Lipová and Prenet). Different colors of the branches indicate different phyla. The relative abundance of each genus in each soil is displayed outside the circle with different colors denoting different rhizosphere samples.

The rhizospheric soil samples detected 29,770 bacterial amplicon sequence variants (ASVs) with 99% homology (Excel S3). Among them, 15,835 unique bacterial ASVs were present in the L-site, while the P-site documented 9576 unique ASVs (Figure 2A, Excel S4). The detected ASVs were assigned to 46 bacterial phyla and 2 archaeal phyla. The abundant bacterial phyla include Proteobacteria (L-site 39%; P-site 43%), Acidobacteriota (L-site 25%; P-site 30%), Actinobacteria (L-site 18%; P-site 16%), Verrucomicrobia (L-site 5%; P-site 4%), and Bacteroidota (L-site 3%; P-site 2%) (Figure 2B). The evolutionary tree representing the top 100 abundant bacterial genera includes *Granulicella*, *Bradyrhizobium*, *Acidothermus*, *Rhodonobacter*, *Roseiarus*, *Burkholderia-Caballeronia-Paraburkholderia*, *Occallatibacter*, and *Candidatus Solibacter* (Figure 2B). Similarly, the fungal ITS2 sequencing generated 7570 ASVs, including 3778 ASVs unique to L-site soil, whereas 2537 ASVs were exclusive to P-site (Figure 2C, Excel S5). These ASVs were assigned to 18 fungal phyla, of which Basidiomycota (L-site 53%; P-site 44%), Ascomycota (L-site 35%; P-site 44%), and Mortierellomycota (L-site 4%; P-site 5%) were dominant in the rhizospheric soil from the two sites. The highly abundant fungal species detected in the rhizospheric soil belonged to the fungal genera *Tylospora*, *Macrolepiota*, *Hygrophorus*, *Piloderma*, *Exophiala*, and *Cenococcum,* which were dominant in the L-site. Meanwhile, *Hyaoscypha*, *Archaeorhizomyces*, *Amphinema*, and *Amanita* were prevalent in the P-site (Figure 2D).

#### 2.3.3. Alpha Diversity (α)

**Figure 3:**
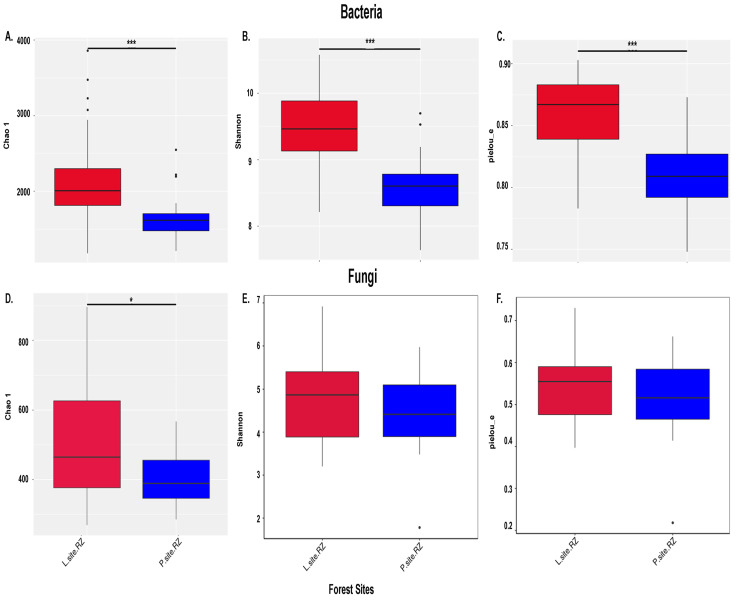
Alpha diversity indices. (**A**) Chao1 index representing bacterial richness. (**B**) Shannon index estimating the bacterial diversity. (**C**) Pielou index representing bacterial evenness. (**D**) Fungal richness (Chao1 index). (**E**) Diversity of fungal communities estimated by Shannon index. (**F**) Pielou index indicating the fungal evenness in the rhizosphere samples from two seed orchards, Lipová (L-site) and Prenet (P-site). The level of significance was determined using the Wilcox test (“*” denotes *p* < 0.05 and “***” indicates *p* < 0.001).

The completeness of the amplicon sequencing approach determined by Good’s coverage index (>99%) indicated that the sequencing depth covered the majority of the taxa present in the samples, and only <1% of the microbial diversity could not be recovered. Similarly, the rarefaction curves tend to reach a plateau, indicating the coverage of the whole microbial diversity in the soil samples by the Illumina sequencing (Figure S2). The bacterial diversity (Shannon index, L-site 11.0; P-site 9.66), community richness (Chao1, L-site 20,460; P-site 14,308), and community evenness (Pielou index, L-site 0.77; P-site 0.70) were significantly higher in the L-site (Wilcox test, *p* < 0.001) (Figure 3A–C). Similarly, the fungal community richness (Chao1, L-site 5184, P-site 3949, Wilcox test, *p* < 0.05) was substantially higher in L-site, while the fungal diversity (Shannon Index, L-site 7.08; P-site 6.994) and community evenness (Pielou-L-site 0.576; P-site 0.589) did not differ significantly (Figure 3D–F).

#### 2.3.4. Beta Diversity (β)

**Figure 4:**
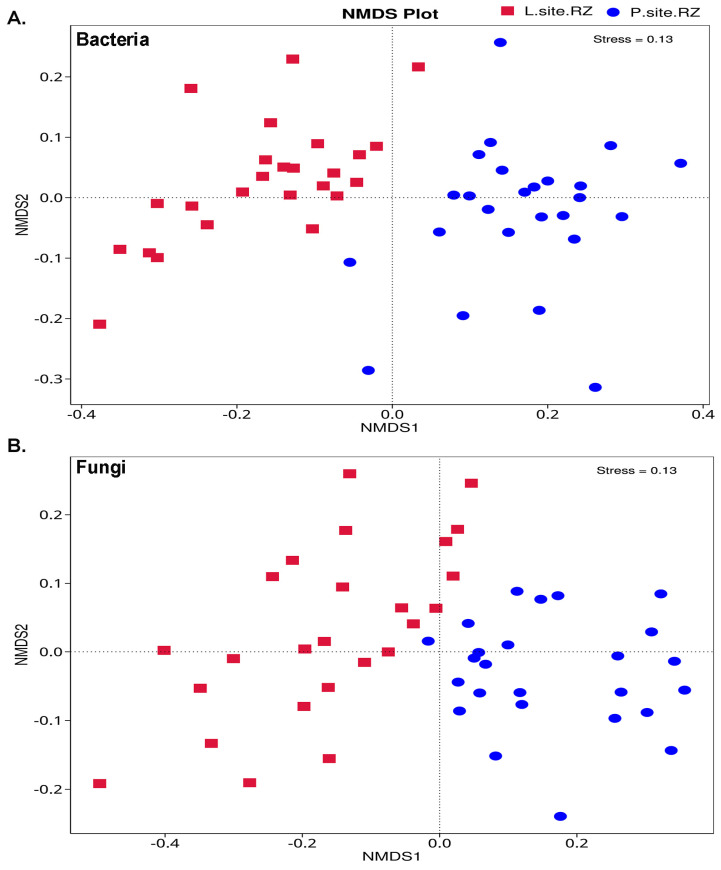
Non-metric multi-dimensional scaling analysis (NMDS) displaying (**A**) the difference in the bacterial communities in Lipová and Prenet rhizospheres. (**B**) The extent of variation in the fungal communities present in the soil samples. The data points in the same color represent soil samples from the same site. Different symbols designate the soil samples from different locations.

The total microbial diversity among the soil samples estimated based on unweighted UniFrac distances distinctly clustered the bacterial communities in the rhizospheric soil samples from the two Norway spruce seed orchards, suggesting the influence of the environment on the soil bacterial population (Figure 4A). Similarly, the fungal population between the two soil samples was clustered in two groups. However, the clusters were close, indicating a lesser impact of site-specific environmental factors on the fungal community structure than the bacterial population (Figure 4B). Consequently, the ADONIS and ANOSIM analyses depicted substantial differences in the microbial diversity between the soil samples from the two sites (Table 1 and Table S2). Hence, such a remark on the influence of the environment on soil microbiome needs further validation.

**Figure 5:**
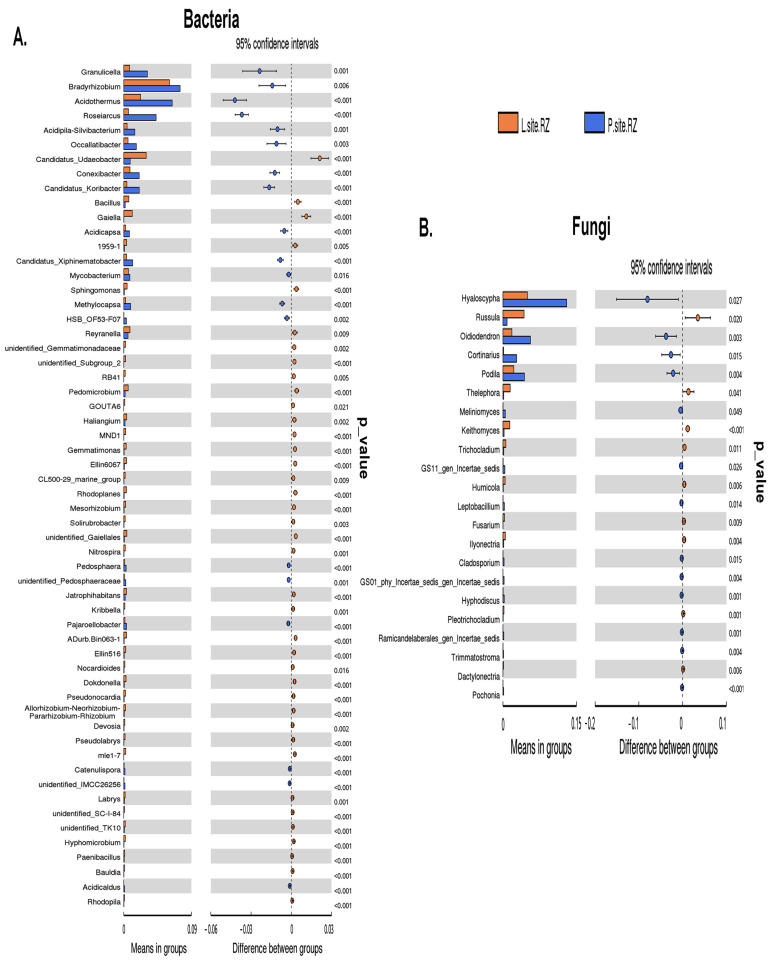
*t*-test analysis to determine the significant variation of (**A**) bacterial and (**B**) fungal communities at the genus level in Lipová and Prenet soils. The last panel denotes the abundance of the genera that significantly differs between the two rhizosphere soils. Each bar represents the mean value of the abundance at the genus level in soil that is significantly different. The right panel denotes the confidential interval between the rhizospheres of the two sites. The left-most part of each circle stands for the lower 95% confidential interval limit, while the right-most part is the upper limit. The center of the circle stands for the difference in the mean value. The color of the circle resembles the soil sample, whose mean value is higher. The right-most value is the *p*-value of the significance test.

*t*-test and MetaStat analysis determined the differentially abundant bacteria belonging to genera *Granulicella*, *Bradyrhizobium*, *Acidothermus*, *Roseiarcus*, *Acidipila*, *Occallatibacter*, *Conexibacter*, *Candidatus Koribacter*, *Candidatus Xiphinematobacter*, and *Acidisoma*, *Agathobacter*, etc. were prevalent in P-site (Figure S3 and Figure 5A). While *Candidatus Udaeobacter*, *Pseudolabrys*, *Gaiella*, *Bacillus*, *Haliangium*, *Rhodoplanes*, *Pedomicrobium*, *Sphingomonas*, and *Reyranella* were dominant in L-site (Figure 5A). Similarly, the significantly abundant fungal genera, including *Russula*, *Thelephora*, *Keithomyces*, *Trichocladium*, *Ilyonectria*, *Fusarium*, *Achroistachys*, *Xanthothecium*, and *Gamsia,* were significantly abundant in L-site (Figure 5B and Figure S4). While *Hyaloscypha*, *Oidiodendron*, *Cortinarius*, *Podila*, *Meliniomyces*, and *Filobasidiella* were predominant in the P-site (Figure 5B and Figure S4).

**Figure 6:**
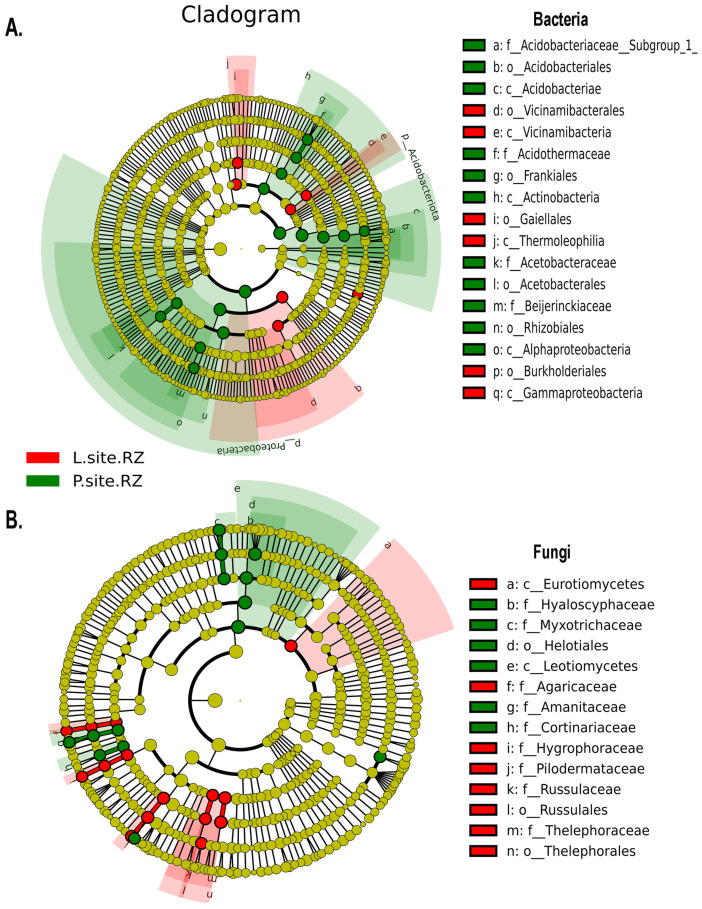
LEfSe [linear discriminant analysis (LDA) Effect Size] analysis indicating the differentially represented microbial biomarkers in the two Norway spruce seed orchards (L, P). (**A**) The cladogram illustrates the presence of bacterial communities that are significantly different between the two soil samples. (**B**) The cladogram represents the fungal biomarkers in the rhizosphere of two sites. The circles radiating from inside to outside denote the taxonomic level from phylum to genus. Each circle represents a distinct taxon at the corresponding taxonomic level. The size of each circle is proportional to the relative abundance of each taxon. Bacterial and fungal biomarkers with significant differences are colored according to the color of the corresponding soil samples, whereas yellowish-green circles resemble non-significant species. Red and green nodes indicate that these species contribute highly to the group. The letters above the circles describe the different biomarkers.

Furthermore, the bacterial biomarkers determined by the Linear discriminant analysis effect size (LEfSe) with LDA score [log10] > 4 showed the presence of significantly abundant and consistent bacterial population belonging to members from class Alphaproteobacteria (order—Rhizobiales, family—*Beijerinckiaceae* and order—Acetobacterales, family—*Acetobacteraceae*), Acidobacteriae (order—Acidobacteriales, family—*Acidobacteriaceae_Subgroup_1*), and Actinobacteria (order—Frankiales, family—*Acidothermaceae*) in P-site, while class Gammaproteobacteria (order—Burkholderiales), Vicinamibacteria (order—Vicinamibacterales), and Thermoleophilia (order—Gaiellales) were the biomarkers from L-site soil (Figure S5A and Figure 6A). Alternatively, the members belonging to the fungal class Leotiomycetes (order—Helotiales, family—*Hyaloscyphaceae*); family—*Myxotrichaceae*, *Amanitaceae*, *Cortinariaceae* were the fungal biomarkers in the soil samples collected from P-site (Figure S5B and Figure 6B). While the fungal species from class—Eurotiomycetes; order—Thelephorales (family—*Thelephoraceae*), Russulales (family—*Russulaceae*), family—*Agaricaceae*, *Hygrophoraceae*, *Pilodermataceae* were the biomarkers in L-site (Figure S5B and Figure 6B).

#### 2.3.5. Functional Composition

**Figure 7:**
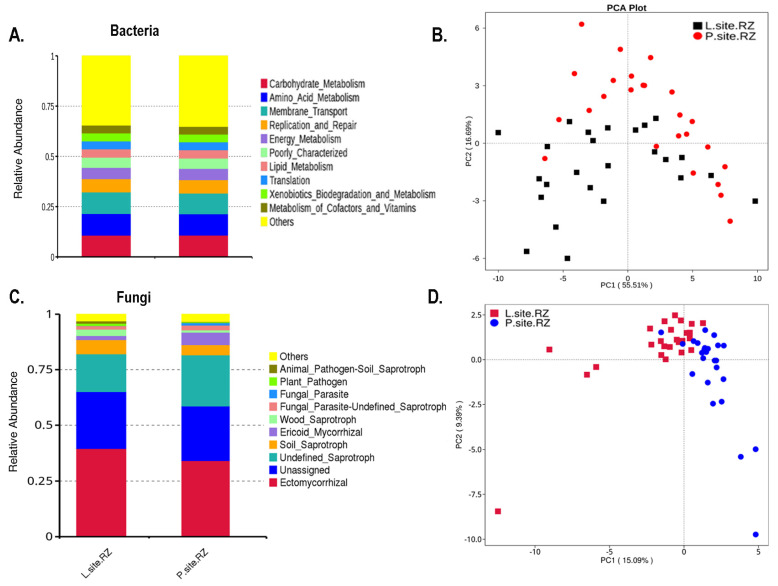
(**A**) Barplot representing the relative ASV abundance contributing to the top 10 gene functions in the rhizospheric soil. “Others” represents the relative ASV abundance for the rest of the gene functions. (**B**) PCA plot shows overlap in the predicted functional contribution of soil bacterial communities in two sites (Lipová and Prenet) based on PICRUSt2 analysis. (**C**) Barplot representing the relative ASV abundance contributing to the top 10 fungal guilds in the rhizospheric soil. “Others” represents the relative ASV abundance for the rest of the ecological guilds. (**D**) PCA plot shows an overlap in the predicted ecological guilds of the fungal population in two sites (Lipová and Prenet) based on FUNGuild analysis.

The putative metabolic function of the bacterial communities in the rhizospheric soil was predicted based on the relative abundance of the bacterial 16S ribosomal gene sequences where the ASVs are mapped to the KEGG database (Excel S6). The bar plot represents the top 10 predicted functions assigned to the bacterial communities in soil, including carbohydrate metabolism, amino acid metabolism, membrane transport, lipid metabolism, metabolism of cofactors and vitamins, degradation, and metabolism of xenobiotics that did not differ significantly between the soil samples from two seed orchards, suggesting these functions are consistent in the soil bacterial communities (Figure 7A). Moreover, the PICRUSt2 (v2.3.0) data represented by the PCA plot revealed that the predicted bacterial functional gene composition between the rhizospheric soil from the two sites was similar, and there was no distinct clustering based on the functions (Figure 7B). The *t*-test analysis documented the significantly abundant functions based on the KEGG database in the rhizospheric soil samples (Figure S6).

Similarly, the ecological guild of the fungal population having similar functions was predicted by FUNGuild software v1.0, which is used to taxonomically analyze the ASVs based on their functions (Excel S7). The rhizospheric soil samples from both sites were dominated by ectomycorrhizal fungal population (Figure 7C). The PCA plot representing the overall fungal population based on their functions demonstrated an overlap between them, suggesting similar functional potential of the fungal populations in the two sites (Figure 7D). Interestingly, the *t*-test analysis revealed a significantly high abundance of Ericoid mycorrhiza in the P-site, while wood saprotrophs were highly prevalent in the L-site (Figure S7). However, it is worth mentioning here that FUNGuild and PICRUSt 2 are prediction software and might not imply the actual functional role of the microbial population. Hence, further experiments are needed to validate the functional role of these microbial communities.

#### 2.3.6. Microbial Co-Occurrence Network

**Figure 8:**
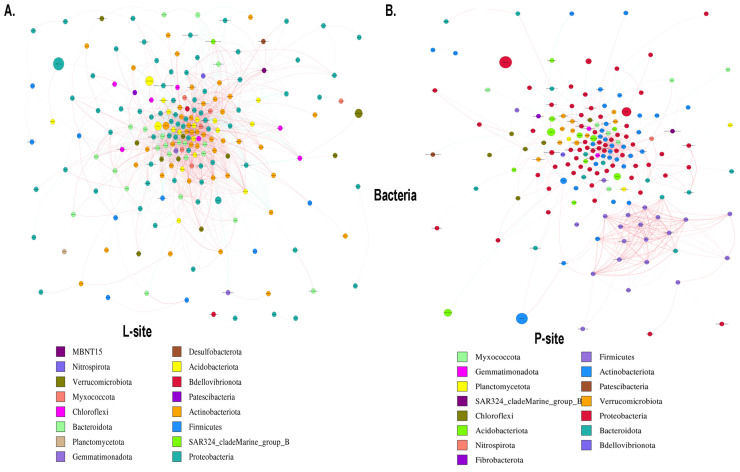
Co-occurrence network analysis illustrating the interaction between the bacterial communities within (**A**) L-site (**B**) P-site. The connections between the nodes indicate significant correlations (Spearman’s correlation coefficient cutoff = ±0.6, *p* < 0.05). The size of each circle is proportional to the relative abundance of each taxon, and different colors of the nodes indicate different phyla.

**Figure 9:**
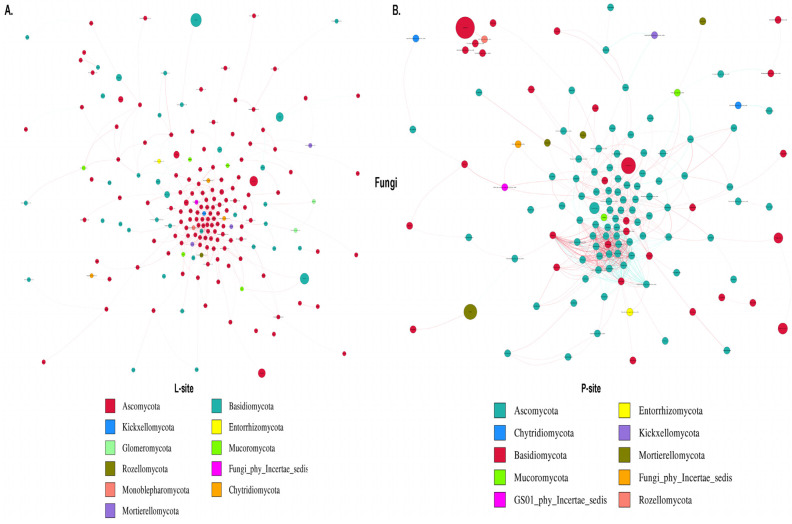
Co-occurrence network analysis illustrating the interaction between the fungal communities within (**A**) L-site (**B**) P-site. The connections between the nodes indicate significant correlations (Spearman’s correlation coefficient cutoff = ±0.6, *p* < 0.05). The size of each circle is proportional to the relative abundance of each taxon, and different colors of the nodes indicate different phyla.

The co-occurrence network analysis assists in understanding the complex microbial interactions and their responses to climate change. Bacterial network analysis represented the co-occurrence of dominant bacterial species in two sites with different precipitation regimes (Figure 8). The rhizosphere soil bacterial network comprises 227 nodes and 3744 edges with significant correlation in the L-site (Figure 8A). Among them, 2896 edges showed a positive correlation coefficient, while 848 edges represented a negative correlation coefficient (Excel S8). The major bacterial nodes in L-site belonged to Proteobacteria (37.4%), Actinobacteriota (21.14%), Bacteroidota (10.13%), and Acidobacteriota (7.92%). On the contrary, the bacterial co-occurrence network was simple in the P-site, representing 192 nodes and 1658 edges with 1402 positive interactions, while 256 edges constituted negative interactions (Figure 8B, Excel S8). P-site documented the highest positive bacterial interactions (~84%), with the major nodes belonging to Proteobacteria (35.93%), Actinobacteriota (18.75%), Firmicutes (12.5%), and Bacteroidota (6.77%). The network diameter was 10 for both sites. The average network distance (L-site 2.77; P-site 3.52), clustering coefficient (L-site 0.48; P-site 0.51), network density (L-site 0.07; P-site 0.04), average degree (L-site 33.13; P-site 17.27), and the modularity index (MD) (L-site 0.27; P-site 0.46) were estimated, representing the complexity of the bacterial population in the rhizospheric soil between the two sites. The higher modularity value documented in the P-site bacterial network indicates a modular community structure where the nodes are densely connected within the communities to form modules with similar ecological niches, suggesting a less complex network. Similarly, the fungal network analysis revealed 227 nodes and 708 edges (692 positive and 16 negative interactions) with significant correlation in L-sites (Figure 9A, Excel S9). In contrast, the P-site rhizospheric fungal co-occurrence network documented 185 nodes and 1280 edges (Figure 9B, Excel S9). Among the fungal interactions, 874 were positive, while 406 were negative. The major fungal nodes include Ascomycota (L-site 66.07%; P-site 60%) and Basidiomycota (L-site 22.46%; P-site 29.18%) in both sites. Although most fungal interactions were positive, L-site documented the highest positive interactions (>97%). The network diameter (L-site 14; P-site 9), average network distance (L-site 4.62; P-site 3.21), clustering coefficient (L-site 0.33; P-site 0.62), network density (L-site 0.01; P-site 0.04), and average degree (L-site 6.23; P-site 13.83) represented the co-occurrence of fungal communities and their interactions in the rhizospheric space between the sites. In contrast to the bacterial community structure, the fungal community in the L-site documented a higher modularity index (L-site 0.48; P-site 0.34), representing a less complex fungal community structure. The higher average degree of fungal co-occurrence network in the P-site indicates higher interaction with the neighboring fungal nodes. On the other hand, the L-site documented increased interactions with the neighboring bacterial nodes. Such observation suggests that variation in the precipitation regime influences fungal and bacterial populations differently.

## 3. Discussion

The “root-soil-microbe” triangle is regarded as the most dynamic underground interaction in nature and is crucial in maintaining biodiversity in many ecosystems. Such dynamic interactions and feedback between plants and microbes are intensified in the root region (i.e., rhizosphere) [33]. Anthropogenic activities significantly influence global change and disrupt ecosystem functioning. One of the primary consequences of climate change is the alteration of the precipitation regime [34] that profoundly impacts the soil microbial community structure [35]. The present study explores the rhizosphere microbiota (microbial interaction “hot spot”) of two clonal Norway spruce seed orchards under different precipitation regimes. The two Norway spruce stands constituted of genetically identical grafted Norway spruce clones. The precipitation difference between the study sites resulted in intriguing differences in soil metabolite content, selected elements, and overall microbial composition, aiding a greater understanding of climatic influence on forest tree rhizosphere microbiota.

Soil water content is essential to soil processes and belowground microbial activity. Water availability enhances microbial activity, promotes organic matter decomposition, and decreases carbon sequestration in soil [36,37]. Precipitation events are closely associated with the soil water content and regulating the organic matter turnover in soil [19,38]. Our previous study on the bulk soil from these two sites suggested that the high soil moisture content due to increased precipitation resulted in higher organic matter content and total nitrogen in the P-site [39]. Although precipitation changes can influence global variations in soil pH [40], our earlier findings revealed no significant difference in soil pH and conductivity between these two Norway spruce seed orchards [39]. Variation in the precipitation pattern influences the soil microbiota [35,41], impacting nutrient cycling and metabolite content [42]. Metabolites play a crucial role in stabilizing carbon in soil. Soil metabolites, such as fatty acids, amino acids, lipids, organic acids, sugars, and volatile organic compounds, are closely connected to soil biogeochemical cycles driven by soil microorganisms [43]. Microbial carbon use efficiency is regulated by substrate diffusional limitations associated with soil water content [44]. Hence, the soil moisture content is pivotal in regulating soil microbial activity and, in turn, impacts the soil metabolite content [44].

The present study documented an increased abundance of rhizosphere soil metabolites belonging to fatty acids, alcohols, acids, and terpenes in the P-site, while carbohydrates were abundant in the L-site. Such abundance in the metabolite profile could be correlated with the higher abundance of Proteobacteria, Acidobacteriota, Actinobacteriota, Bacteroidata, and Firmicutes, as these bacterial phyla exhibit a crucial eco-physiological role in the cycling of essential elements such as nitrogen, carbon, and sulfur [45]. Interestingly, the high abundance of fatty acids and terpenes could be co-related with the lower abundance of Actinobacteriota and Chloroflexi in the P-site [46]. Similarly, Shi et al., 2011 reported that adding carbohydrate metabolites to forest soil significantly increased the relative abundance of some dominant bacterial taxa, such as Actinobacteriota, Proteobacteria, and Firmicutes [47]. This corroborates our findings, suggesting the importance of carbohydrates in modulating the soil bacterial community structure by boosting the abundance of selective microbial communities. One logical explanation can be that these bacterial communities better utilize carbohydrates as a carbon source than others, which gives them a selective advantage. However, such a correlation needs further experimental validation. It is worth mentioning here that the rhizosphere chemistry is complex and undergoes continuous alterations with the constant flux of metabolites from the root exudates and the microbial secretions [48]. The soil metabolites are attributed to the soil organic matter, plant exudates, and microbial metabolites [32]. In the current study, it was impossible to independently differentiate the contribution of these driving factors to the soil metabolite profiles. The mechanism behind how the complex metabolite pools are related to the microbial diversity in the rhizospheric space is still unclear and needs further investigation. Understanding the metabolite profiling in the rhizospheric soil will provide insights into how the plants and the belowground microbes interact in response to environmental changes [49].

The microbial diversity and functioning are often influenced by moisture content in the soil. In the current study, comparatively higher soil moisture content in the P-site resulted in lower bacterial diversity. However, the fungal diversity did not alter with differences in the soil water content. A similar observation was reported by Yang et al. 2021, which showed that variation in precipitation strongly affected the soil bacterial communities but not fungi in a meadow grassland in northeastern China [50]. The response of soil bacteria and fungi differs with the variation in soil water availability. Bacterial response to soil moisture content is faster due to different physiological characteristics and survival strategies than fungi [51]. Moreover, increased soil water content due to higher precipitation might result in osmotic stress, causing a decrease in bacterial diversity in P-site [52]. The rhizospheric fungal diversity did not differ between the two sites with different precipitation regimes, suggesting stable and resistant fungal populations where the fungal hyphae are better adapted to the low moisture content in the soil while reaching out for nutrient resources [53].

The present study documented a high abundance of Proteobacteria, Acidobacteriota, including *Granulicella*, *Roseiarcus*, *Acidobacteria*, and *Acidipila* in the P-site rhizosphere. These bacterial members play an important role in the decomposition of organic matter and increase the availability of nitrogen, calcium, and phosphorus in acidic soil [54,55]. The high abundance of acidophilic bacteria might be correlated with the high abundance of fatty acids and organic acid metabolites in the P-site soil [56]. Bi et al., 2022 reported that a high abundance of fatty acids contributes to soil acidity and significantly alters the conifer root microbial community structure [56]. Consequently, our previous study on bulk soil from the two sites already documented low pH in the P-site compared with L-site [39]. However, the bulk soil pH between the two sites was not significantly different. The high abundance of *Bradyrhizobium* in the P-site is reported to play an important role in nitrogen fixation and nutrient acquisition (N and P) and interacts with mycorrhizal fungi [57]. This can be correlated with the high abundance of total nitrogen present in the soil samples from the two sites [39]. Soil moisture and temperature drastically affect fungal growth and symbiotic functioning [58,59]. High soil moisture and low temperature in the P-site may provide a favorable environment for mycorrhizal development. Studies reported that precipitation can significantly alter arbuscular mycorrhizal fungi (AMF) communities by modifying their interactions among different AMF groups [60]. Furthermore, the dominance of the mycorrhizal community in the P-site might be due to their association with different “Mycorrhizae Helper Bacteria” (MHB) that promote and stimulate the establishment of mycorrhizal symbiosis with the host plant [61]. Such bacterial genera, including *Azospirillum*, *Burkholderia*, *Bradyrhizobium*, *Pseudomonas*, *Rhizobium*, *Bacillus*, *Brevibacillus,* etc., were observed in the rhizosphere microbiota. Additionally, a high abundance of *Acrodontium*, *Oidiodendron*, and *Cortinarius* was documented in the P-site rhizosphere. *Acrodontium* is reported to have inhibitory action against powdery mildew pathogens [62], while *Oidiodendron* sp. is an essential contributor to the forest ecosystem that exhibits many plant cell wall degrading enzymes [63] and also participates in mycorrhizal association [64]. *Cortinarius* sp. is one of the most important symbiont mycorrhizal fungi around tree roots of forest ecosystems [65].

Interestingly, the L-site rhizosphere receiving less precipitation compared with the P-site exhibits a higher bacterial diversity. The bacterial genera, including *Candidatus*, *Pseudolabrys*, *Bacillus*, *Gaiella*, and *Sphingomonas*, were the major constituents of the L-site rhizosphere. These bacterial species utilize plant-derived hydrocarbons and polysaccharides and play an essential role in nutrient cycling in the soil [66,67,68,69]. The rhizosphere of the L-site promotes the assemblage of ectomycorrhizal fungi *Russulla* and *Thelephora*. The ectomycorrhizal fungi have been reported to select several helper bacteria in the mycosphere to grow and maintain mycelium in the soil [70,71,72]. Moreover, a significant abundance of pathogenic fungi, including *Ilynocteria*, *Fusarium*, *Trichocladium*, and *Metarhizium*, were documented at the L-site. *Metarhizium*, an entomopathogenic fungi, protects plants from insect attacks [73], while *Ilynocteria*, *Fusarium*, and *Trichocladium* are plant pathogenic fungi [74,75,76]. Interestingly, the low abundance of pathogenic fungal communities in P-site compared with L-site might be due to the presence of *Occallatibacter*, which is reported to hydrolyze chitin, protecting plants from pathogenic fungal attacks [67]. However, such interpretations require further experimental validation. Among other significantly abundant fungi in the L-site, *Humicola* is reported to possess β-glucosidase gene (*bgl4*) and β-xylosidase gene (*hxylA*) coding for β-glucosidase and β-Xylosidases and might be responsible for cellulose degradation [77,78]. The rhizosphere soil biomass mainly consists of plant materials constituting hemicellulose, cellulose, lignin, pectin, and proteins. Cellulose-degrading microbial communities in soil play an important role in nutrient cycling and organic matter decomposition [79].

The putative functional prediction documented by PICRUSt2 showed no distinct clusters in the overall putative functions of the bacterial communities between the two sites, suggesting similar functional potential of the rhizospheric soil bacterial communities from the two seed orchards. Similarly, the ecological guild in the two sites overlapped, indicating a lesser impact of precipitation change on the fungal population. However, such predictions are based on the predicting software and might not depict the actual scenario. Hence, further functional validations are needed to confirm such observations. Furthermore, microorganisms flourish through complex association networks rather than in isolation. The highly connected species potentially play a vital role within the microbial community [80]. In the bacterial network, the species belonging to the bacterial phyla Proteobacteria, Actinobacteriota, Acidobacteriota, Bacteroidota, and Firmicutes showed significantly more connections than other phyla. These bacterial genera might be necessary for biogeochemical cycling and carbon mineralization in forest ecosystems [81,82,83]. Interestingly, these major bacterial nodes have different growth rates; for instance, some of the taxa belonging to Proteobacteria are fast growers, while bacterial genera belonging to Acidobacteria are slow growers [84,85]. Interactions between these nodes suggest that the diverse microbial pool maximizes the resources through their interactions and maintains forest ecosystem functioning [80]. In the fungal co-occurrence network, Ascomycota and Basidiomycota formed a significant share of the connections. The fungal genera belonging to these phyla are primarily responsible for the decomposition of lignin and cellulose, facilitating the decomposition of organic matter and promoting soil nutrient cycling within forest ecosystems [86]. The overall microbial interactions were higher in the L-site compared with the P-site. In particular, the bacterial interactions were more than twice in the L-site than in the P-site. Although the number of microbial interactions was lower in the P-site, the higher clustering coefficient of the microbial network suggested that the microbial community structure in the P-site is highly interconnected and tends to close clusters. However, the functional relevance underlying such interpretations needs further experimental endorsements.

## 4. Materials and Methods

### 4.1. Site and Sampling

The soil samples were collected from the rhizosphere of Norway spruce clonal trees from two spruce seed orchards in the Czech Republic. The two sites, namely Prenet (P) (49.2354172 N, 13.2112808 E, 970 m a.s.l., average temp. 7.044 °C, avg precipitation 1306.48 mm) and Lipová Lhota (L) (49.2816108 N, 13.5515606 E, 560 m a.s.l., average temp. 8.644 °C, avg precipitation 633 mm), consisted of five Norway spruce clonal tree varieties (1901, 1902, 1908, 1941, and 1950). Each of these grafted clonal tree varieties had five tree replicates (Table S1). The rhizospheric soil samples were collected at a depth of 15 cm for each clonal tree replicated from the two sites. However, as these are grafted trees with different roots, we did not consider the differences in the rhizosphere microbiome between the clonal tree varieties. Five replicates of rhizosphere soil samples for each clonal tree variety were randomly collected at a distance of 20–30 cm from the tree trunk and ~10 mm from the roots. A total of 50 soil samples (25 samples from each site) were collected in sterile plastic ziplock bags, brought to the laboratory, and sieved through a 2.0 mm screen sieve. One part was stored at 4 °C for the determination of soil properties, and the remaining soil samples were stored at −80 °C for soil metabolite profiling and DNA extraction to determine microbial community structure using amplicon sequencing targeting the bacterial 16S rRNA gene and fungal ITS2 region.

### 4.2. Soil Texture, Moisture Content, and Trace Elements

The soil texture was determined by laser granulometry (CILAS 1190 LD) in wet mode to measure the size of the soil particles ranging from 0.04 to 2500 μm. The soil texture analysis was done as described by [87]. The soil texture determination was based on three soil fractions: clay (>2 µm), silt (2–63 µm), and sand (63–2000 µm) [88]. The water content of the rhizospheric soil was estimated by drying 10g of soil at 105 °C in an oven for 24 h. Furthermore, the elements (Al, Ca, Fe, K, Mg, Mn, Na, P, S, Si, Zn) present in the rhizospheric soil were determined following the Mehlich 3 extraction procedure [89]. The Mehlich 3 extraction solution consisted of 0.2M acetic acid, 0.015M ammonium fluoride, 0.013M nitric acid, 0.001M EDTA, and 0.25M ammonium nitrate at pH 2.5. The air-dried soil was extracted with 1:10 (*m*/*v*) soil: Mehlich 3 solution for 10 min, and the extract was measured by ICP-OES and measured using ICP EOS Agilent 5100 [90]. Rhizospheric soil samples from three clonal varieties for each site were tested for the soil moisture content, soil texture, and the amount of selected elements present. All the results were expressed based on the dry weight of the soil. The student’s test is performed to determine the level of significance (*p* < 0.05).

### 4.3. Metabolomic Profile

The rhizospheric soil metabolite profiling was performed as described in our previous study [39]. A total of 500mg of freeze-dried homogenized rhizospheric soil samples was added to 600 µL of methanol:H_2_O, 3:1 (*v*/*v*) mixture. An equal volume of ethyl acetate (600 µL) and 10 µL of internal standard adonitol (0.5 mg/mL, Internal standard A, IS_A) was added to the soil mixture, sonicated for 30 s, and incubated at 10 °C for 15 min at 2000 rpm, followed by centrifugation at 4 °C for 15 min at 16,000× *g*. The supernatant was collected and vacuum-dried using a vacuum concentrator (Module 4080C, Hanil Science Industrial, Gimpo, Republic of Korea) and resuspended in anhydrous pyridine (50 µL) and methoxyamine hydrochloride in pyridine (25 mg/mL, 50 µL), followed by an incubation for 90 min at 40 °C at 1700 rpm. After incubation, N, O-Bis(trimethylsilyl)trifluoroacetamide with trimethylchlorosilane (BSTFA + TMSC, 100 µL) was added and incubated at 40 °C for 30 min. To this, 10 µL of internal standard B (1-bromoeicosane, 0.52 mg/mL in hexane) was added and centrifuged for 5 min at 3000 rpm. The supernatant was carefully transferred and analyzed on two-dimensional comprehensive gas chromatography with mass detection (GCxGC-MS; Pegasus 4D, Leco Corporation, St. Joseph, MI, USA) controlled by ChromaTOF v4.5. GCxGC analyses were carried out using a combination of polar and non-polar separation columns with the standardized parameters as described in our earlier study [39]. The trimethylsilyl derivatives of the metabolites were normalized according to the amount of the sample during extraction and the internal standards. After normalization, the metabolites were identified based on the NIST Library, Fiehn Library, and in-house-built mass library. The data were represented by Sparse PLS discriminant plot based on sPLS-DA algorithm [91]. The statistical evaluation of the normalized data was carried out by MetaboAnalyst 5.0 (www.metaboanalyst.ca (accessed on 16 September 2020)) [92]. Analysis of variance (ANOVA) and *t*-tests were used to evaluate the statistical significance of the metabolites present in the sample.

### 4.4. DNA Extraction, Amplification, and Sequencing

The rhizospheric soil DNA (250 mg) was extracted using the Nucleospin soil DNA purification kit (Macherey Nagel, Dueren, Germany) following the manufacturer’s protocol. The isolated soil DNA was quantified on a Qubit 2.0 Fluorometer using a Qubit 2.0 high-sensitivity dsDNA assay kit, electrophoresed on 1% agarose gel to check the DNA integrity, and sent for high-throughput amplicon sequencing (Novogene, Beijing, China). The purified DNA was diluted to 1 ng/µL and used as a template for amplification with universal primers tagged with specific barcodes targeting the bacterial 16S rRNA gene (341F/806R, V3-V4 region) [93] and the fungal ITS2 region (ITS3, ITS4) [94]. Phusion High-Fidelity PCR Master Mix (New England Biolabs, Ipswich, MA, USA) was used for PCR amplification. A no-template control was added to the PCR reaction to check for contamination. PCR amplicons were then pooled at equi-density and gel purified (Qiagen Gel Extraction Kit, Qiagen, Hilden, Germany) before library preparation. The sequencing library was constructed using NEBNext Ultra II DNA Library Prep-Kit (New England Biolabs, Ipswich, MA, USA). The constructed library was quantified on Qubit 2.0 Fluorometer (Thermo Scientific, Waltham, MA, USA), and a quality check was performed using Agilent Bioanalyser 2100 system and finally sequenced using Illumina Novaseq 6000 platform to generate 250bp paired-end reads.

### 4.5. Sequencing Data Analysis

#### 4.5.1. Data Filtering

Microbiome analysis was performed in QIIME2 software (version 2022.2) [95]. The Illumina paired-end reads assigned to samples were assembled based on their unique barcodes after the removal of the barcode and primer sequence and merged to get raw reads using FLASH (V1.2.11, http://ccb.jhu.edu/software/FLASH/, (accessed on 16 September 2020)) [96]. The raw tags were then quality-checked using fastp software (version 0.23.0) [97] to obtain high-quality clean tags after the removal of reads with Phred Quality score <30. Finally, chimera detection and removal were performed using VSEARCH software (version 2.7.1) [98] to obtain effective tags for further downstream bioinformatic analysis. DADA2 module [99] in QIIME2 software (version 2022.2) was used to denoise the effective tags, and the sequence abundance of less than 5 reads was discarded to obtain the final ASVs (amplicon sequence variables) [100] and the feature table. Further, the species annotation of each ASV was obtained by comparing the ASVs with the SILVA (Release 138.1, for bacterial sequences) (http://www.arb-silva.de/) [101] or UNITE (version 9.0) (for fungal sequences) (https://unite.ut.ee) [102] databases using the Classify-sklearn module (version 2020.6) [103] in QIIME2 (ver 2022.2) [95].

#### 4.5.2. Alpha Diversity

The microbial diversity and community richness within the samples were estimated by the alpha diversity indices such as Shannon index and Chao1 index [104] in QIIME 2. The Pielou index, representing the evenness of the microbial population, was also determined. The rarefaction curves and the Good’s coverage [105] indicated the completeness of the sequencing of the rhizosphere soil samples from two different sites and were represented using R software (Version 2.15.3; R Core Team, 2013, Vienna, Austria) [106]. The statistical significance of the alpha diversity indices was determined using the Wilcox test to compare the two sites.

#### 4.5.3. Beta Diversity

The difference in microbial diversity [107] between the rhizospheric soil samples collected from two different sites was estimated using the unweighted UniFrac distance [108] calculated by QIIME2 software version 2022.2 [95]. Principal coordinates analysis (PCoA) [109] based on the UniFrac distances was represented in R software, where the samples with similar species composition structures tend to cluster together and vice versa. The significant differences in the overall microbial community structure among the rhizospheric soils between the two soil sites were determined using ADONIS and ANOSIM functions [110] in QIIME2 software. The ADONIS function is a nonparametric multivariate variance test based on the distance matrix to determine the differences between sample groups and estimate the significance of the groups by performing permutation tests [111]. ANOSIM analysis, based on the UniFrac distance matrix, evaluates whether the variation among groups is significantly higher than within groups [112]. The significant differences in the microbial species abundance were determined by the *t*-test [113]. Similary, MetaStat analysis [114] was performed to determine the significant differences in observed species abundance among groups via multiple hypothesis-tests for sparsely-sampled features and false discovery rate (FDR). Furthermore, linear discriminant analysis Effect Size (LEfSe) analysis [115] was performed where the linear discriminant analysis scores (LDA score [log10]) > 4 was set as a threshold to determine the high-dimensional biomarkers and to reveal metagenomic characteristics including genes, metabolites, or taxa to distinguish between two samples. It emphasizes the statistical significance and biological consistency of detecting significant biomarkers and identifying characteristics of abundance.

#### 4.5.4. Functional Prediction

The metabolic function of microbes in the rhizospheric soil samples was predicted by mapping the microbial composition obtained from amplicon sequencing to different databases. The prediction of the bacterial communities present in the soil was determined using PICRUSt2 software (version 2.3.0) (Phylogenetic Investigation of Communities by Reconstruction of Unobserved Stats 2) [116], where the bacterial 16S ASVs abundance was mapped against Kyoto Encyclopedia of Genes and Genomes (KEGG) database [117] to predict different putative functions assigned to the bacterial communities in the soil samples. The abundance of functional annotation for the KEGG database was represented by principal component analysis (PCA). The significant KO (KEGG orthologs) were determined using *t*-test. Similarly, the putative functions of the fungal communities in soil were determined by the FUNGuild annotation tool (version 1.0) (http://funguild.org) that taxonomically parses fungal ASVs by their ecological guild [118] and the differentially abundant fungal guilds were determined by *t*-test.

#### 4.5.5. Network Analysis

The microbial abundance data (ASV table data) were converted to a co-occurrence network and visualized by Graphviz-2.38.0 software to understand the complex interactions among the microbial communities. The network analysis provides information on the impact of environmental factors on microbial communities and the interaction between the dominant species in a specific environment [119]. These dominant species often play an essential role in maintaining the structure and functional stability of the microbial community in the environment. The average relative abundance of microbial species less than 0.005% was filtered out. The correlation coefficient of all the samples was calculated using the Spearman Correlation Coefficient (SCC). The effective connections with correlation coefficient cutoff = ±0.6, *p* < 0.05 were selected to construct the network diagram. Different parameters such as nodes, total links, positive links, negative links, network density, network diameter, average degree, modularity degree, clustering coefficients, and average path length were estimated using the igraph package (version 2.0.2) [120]. The nodes represent the microbial genus, and the links or edges denote the interactions (positive or negative) between the microbial taxa. The network density is calculated to assess the closeness of the overall microbial community. It is the ratio of links of each node to the total number of links. The higher the density, the denser or closer the network. The modularity (MD) determines the structure of the network. It is the measure of the strength of the division of the communities into clusters or modules. The MD value ranges from 1 to −1, and values close to 1 signify a strong community structure [121]. The average degree is the average number of links per node, which signifies the average number of neighbors in the network. The network diameter is the shortest distance between the two distant nodes. Meanwhile, the average path length determines the shortest path between the nodes. The clustering coefficients denote the number of links present as a proportion of the number of links that could exist. Higher clustering coefficients determine tightly connected microbial communities within the network and are associated with network robustness [122,123]. The short average path length and the high clustering coefficients signify high network efficiency and are often known as “small-world” networks [124].

## 5. Conclusions

**Figure 10:**
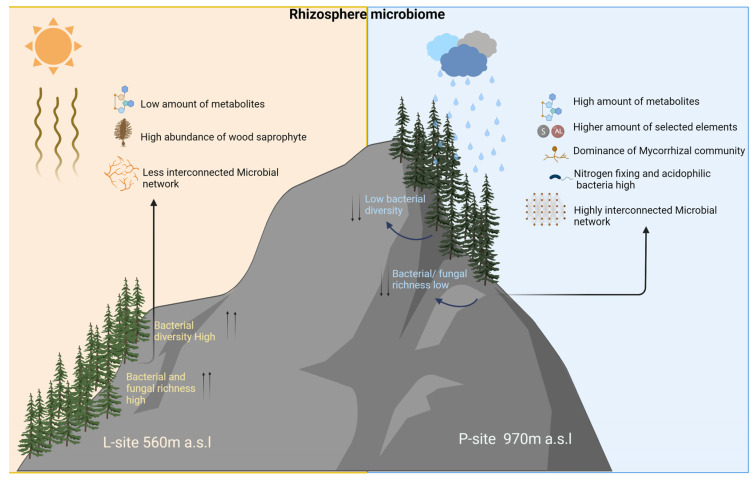
The schematic representation illustrates the impact of long-term precipitation change on the rhizosphere microbiome from two Norway spruce seed orchards.

The current study unveiled the influence of precipitation on microbial assemblage and metabolites in Norway spruce rhizospheric soil (Figure 10). Higher long-term precipitation over three decades impacted the spruce rhizosphere microbiota. The variance in the relative abundance of a distinct microbiome class among the sites indicates climatic interference. The higher moisture content in the P-site receiving higher precipitation resulted in an increase in the amounts of metabolites along with a high relative abundance of acidophilic bacteria, nitrogen-fixing bacteria, and mycorrhizal association. The higher modularity value documented in the bacterial co-occurrence network at P-site indicates a modular bacterial community structure in the rhizosphere, where the nodes are densely connected within the communities to form modules with similar ecological niches, suggesting a less complex network. Nevertheless, the diversity of the fungal community did not vary between the sites, which depicted the lesser impact of precipitation on the rhizosphere mycobiome. Nevertheless, the present study provides an excellent model for studying the effect of climate change on the spruce rhizosphere microbiome and their effect on sustainable forest growth. It will facilitate understanding rhizosphere microbial community dynamics towards future global climate change scenarios and help preserve pristine forests using probiotic formulations.

## Figures and Tables

**Table 1:** ADONIS Analysis. Bray–Curtis method indicates the significant difference between the soil bacterial and fungal communities between the two Norway spruce seed orchards. (Df—degree of freedom, MeanSqs—SS/Df, F. Model—F-test value, R2—the ratio of grouping variance and total variance). Values in parentheses denote Residual Error. The *p*-value represents the significant variation in the microbial community structure.

Diversity	Group	Df	SumsOfSqs	MeanSqs	F.Model	R2	Pr (>F)
Bacteria	L-site vs. P-site	1 (48)	3.74684 (8.50962)	3.74684 (0.17728)	21.13473	0.3057 (0.6943)	0.001
Fungi	L-site vs. P-site	1 (48)	1.45718 (16.17559)	1.45718 (0.33699)	4.32409	0.08264 (0.91736)	0.001

## Data Availability

The datasets presented in this study can be found in online repositories. The names of the repository/repositories and accession number(s) can be found below PRJNA768132 (rhizospheric mycobiome), PRJNA768131 (rhizospheric bacteriome).

## Supplementary Materials

The following supporting information can be downloaded at: https://www.mdpi.com/article/10.3390/ijms25179658/s1.
