# Supplementary material for: Exploring the Rhizospheric Microbial Communities under Long-Term Precipitation Regime in Norway Spruce Seed Orchard
Source: Int J Mol Sci. 2024 Sep 6;25(17):9658. doi: 10.3390/ijms25179658 (PMC11395193; doi:10.3390/ijms25179658)
Supplement: Supplementary file 1 [file ijms-25-09658-s001.zip › Supplementary Material.pdf]

Exploring the Rhizospheric Microbial Communities under Long-Term Precipitation Regime in Norway Spruce Seed Orchard

Dagmar Zádrapová [1,†], Amrita Chakraborty [1,*,†], Petr Žáček [2], Jiří Korecký [1], Anirban Bhar [3] and Amit Roy [1,*]

[1] Faculty of Forestry and Wood Sciences, Czech University of Life Sciences, Kamýcká 129, Suchdol, 165 21 Prague, Czech Republic
[2] Faculty of Science, Charles University in Prague, BIOCEV, Průmyslová 595, Vestec, 252 42 Prague, Czech Republic
[3] Molecular Plant and Microbiology Laboratory (MPML), Post Graduate Department of Botany, Ramakrishna Mission Vivekananda Centenary College, Rahara, Kolkata 700118, India
* Correspondence: chakraborty@fld.czu.cz (A.C.); roy@fld.czu.cz (A.R.)

Figure S1: Heatmap illustrating the 50 most significant metabolites in the rhizospheric soil samples from two sites (L, P) in replicates. The rhizosphere soil samples from three trees per variety were used, and a total of fifteen replicates from each site were studied. The numbers representing the different metabolites detected and the details can be found in Excel S1.

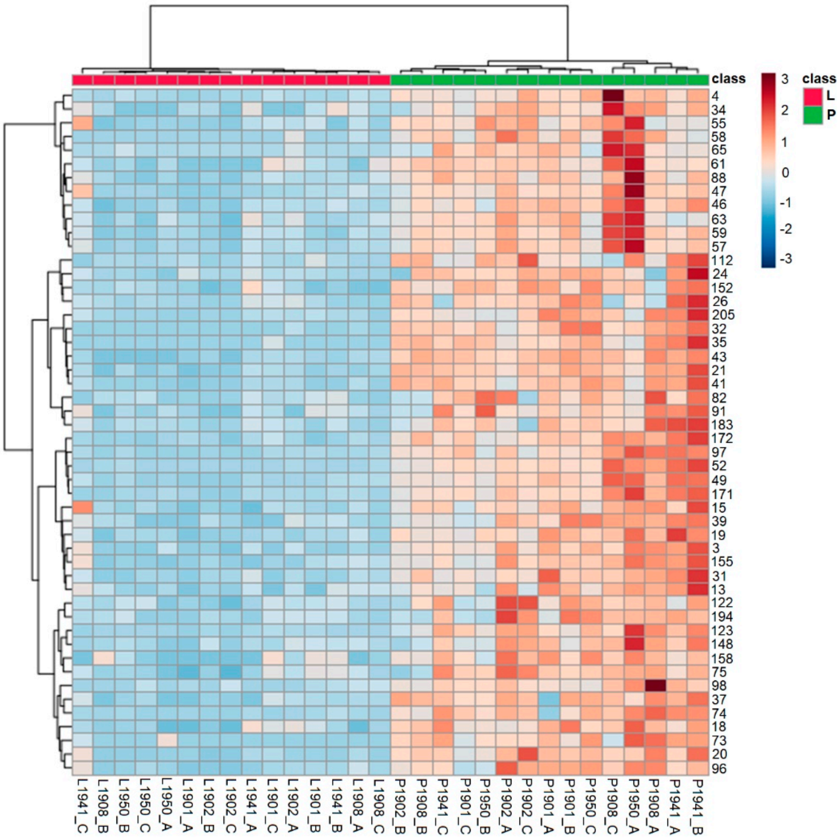

Figure S2: Rarefaction Curves representing observed features (ASVs) for **(A)** bacterial 16S amplicon sequencing (**B**) fungal ITS sequencing of rhizosphere soil samples from two different locations, Lipova (L) and Prenet (P). Different colors and symbols denote different samples.

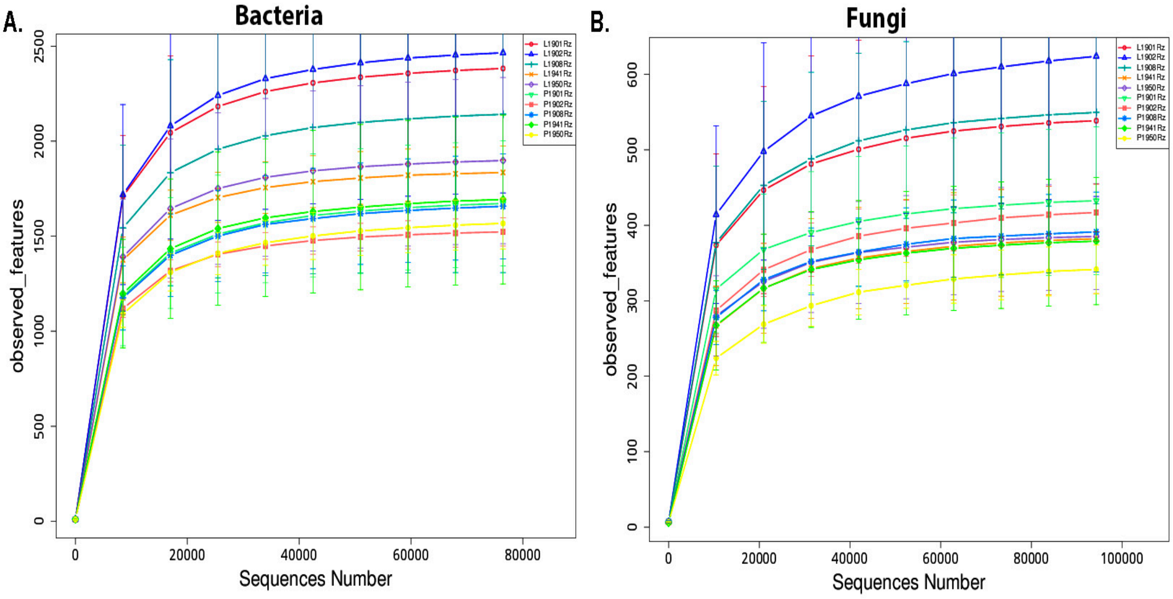

Figure S3: Metastats analysis shows significantly abundant bacterial species in Lipova (L-site. Rz) and Prenet soil (P-site.Rz). The significant differences in the relative abundance are evaluated by the FDR test where the "*" represents significant variation at q value <0.05 while "**" denotes high significance at q value <0.01.

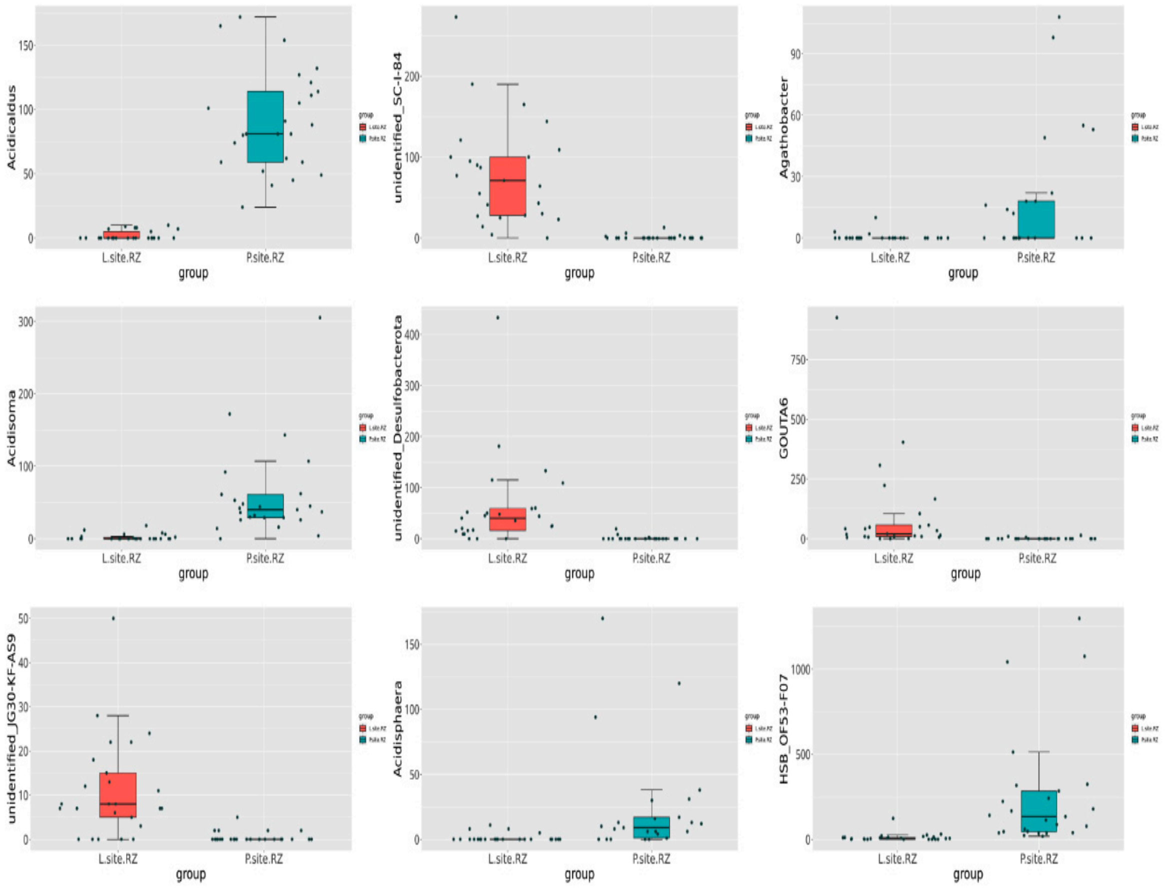

Figure S4: Metastats analysis shows significantly abundant fungal species in in Lipova (L-site. Rz) and Prenet soil (P.site.Rz). The significant differences in the relative abundance are evaluated by the FDR test where the “*” represents significant variation at q value <0.05 while “**” denotes high significance at q value <0.01.

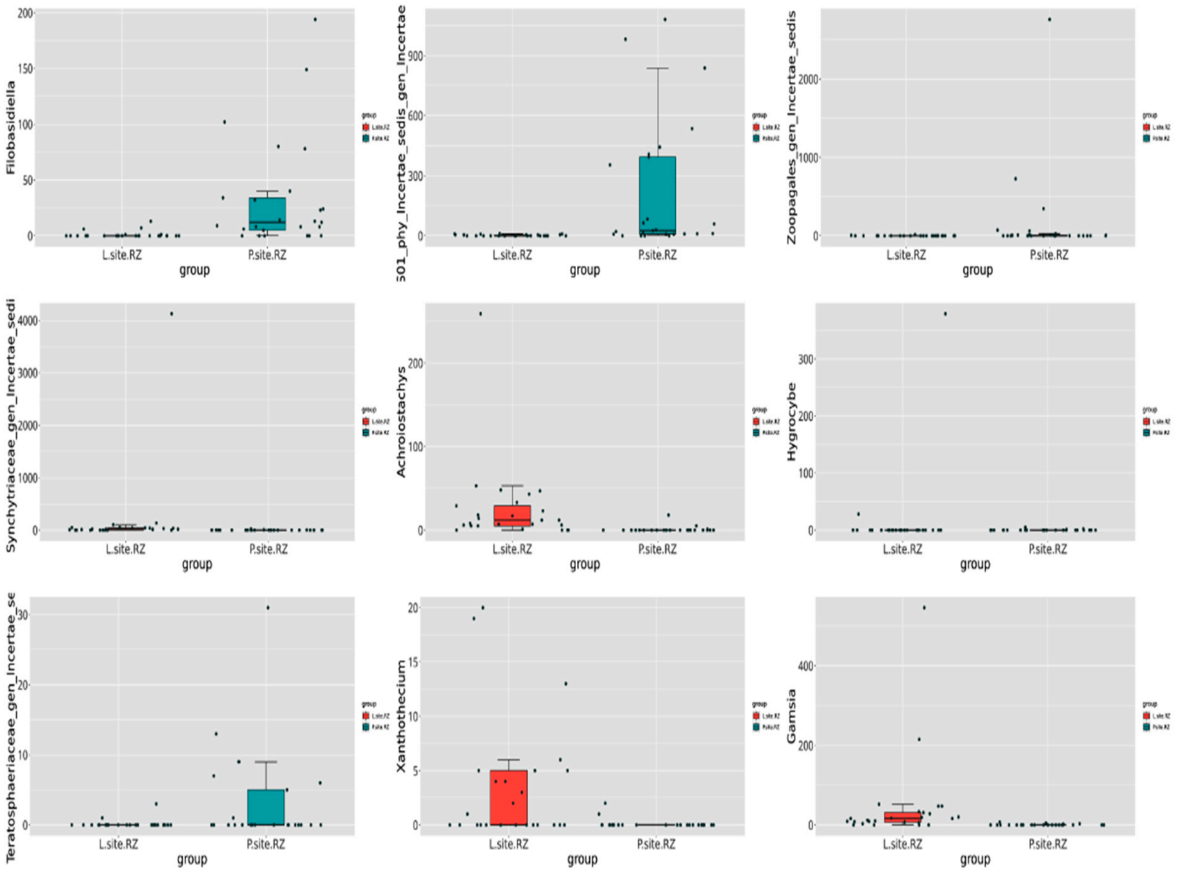

Figure S5: LEfSe analysis representing histogram of the LDA scores illustrating the presence of **(A)** bacterial and **(B)** fungal species (biomarker) whose abundance differs significantly between the soils from Lipova (L.site.Rz) and Prenet (P.site.Rz). The length of each bin, i.e., LDA score, represents the effect size (the extent to which a biomarker can explain the differentiating phenotypes among groups) at the LDA score cutoff threshold >4.

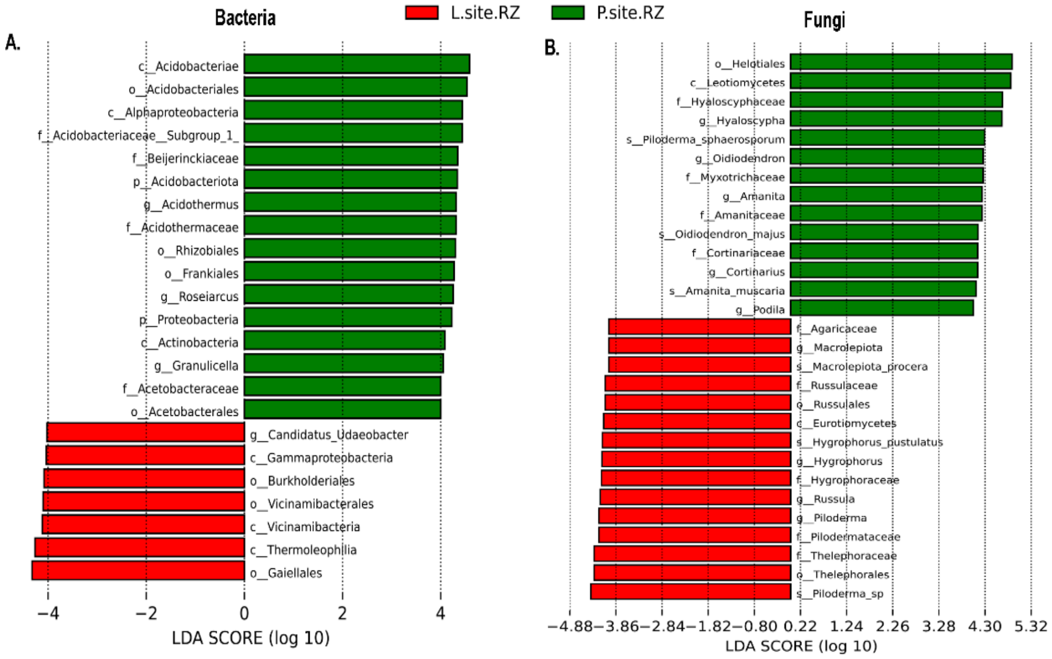

Figure S6: T-test analysis**.** Significant fungal guilds using FunGuild data present in the rhizosphere of Lipova (L.site.Rz) and Prenet (P.site.Rz).. Each bar represents the mean value of the abundance at different ecological guilds, which is significantly different. The right panel denotes the confidential interval between the soils. The left-most part of each circle stands for the lower 95% confidential interval limit, while the right-most part is the upper limit. The centre of the circle stands for the difference in the mean value. The colour of the circle resembles the soil sample, whose mean value is higher. The right-most value is the *p*-value of the significance test.

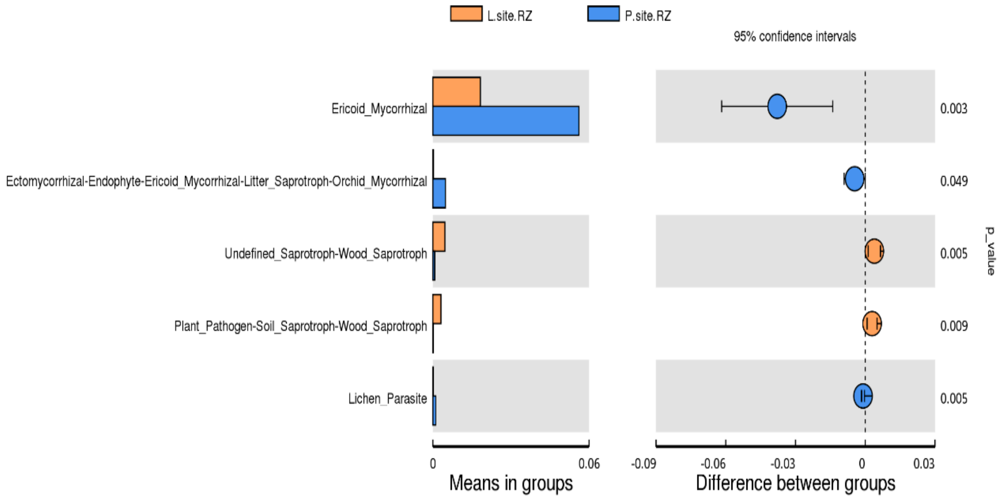

Figure S7: T-test analysis to determine the significant variation of bacterial putative functions based on the KEGG database (KO) using PICRUSt2 data in two soil samples (L.site.Rz and P.site.Rz). Each bar represents the mean value of the abundance of different KO, which is significantly different. The details of KO ID and their relative abundance can be found in Supplementary Excel 6. The right panel denotes the confidential interval between the soils. The left-most part of each circle stands for the lower 95% confidential interval limit, while the right-most part is the upper limit. The centre of the circle stands for the difference in the mean value. The colour of the circle resembles the soil sample, whose mean value is higher. The right-most value is the *p*-value of the significance test.

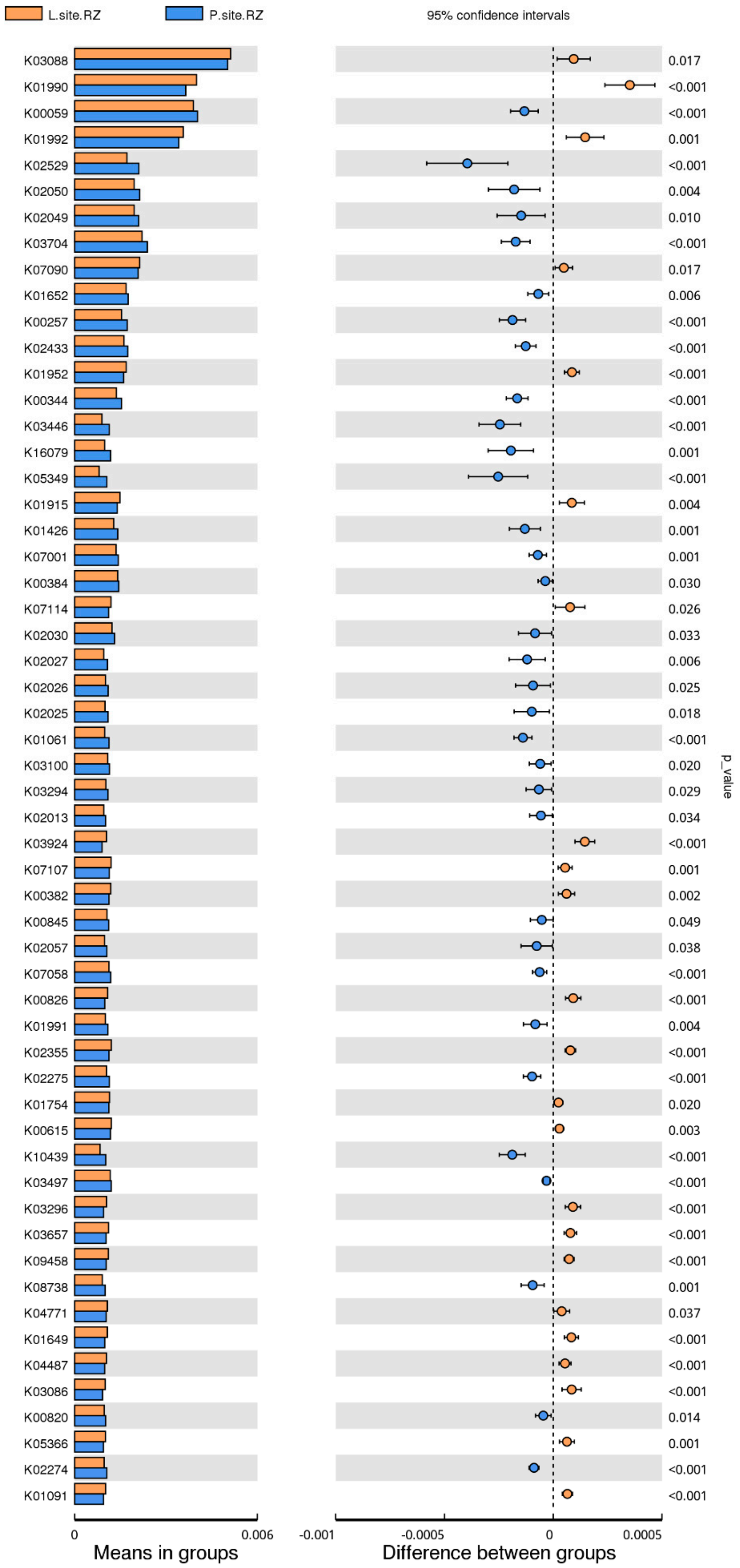

L.site.RZ
P.site.RZ
95% confidence intervals
K03088
K01990
K00059
K01992
K02529
K02050
K02049
K03704
K07090
K01652
K00257
K02433
K01952
K00344
K03446
K16079
K05349
K01915
K01426
K07001
K00384
K07114
K02030
K02027
K02026
K02025
K01061
K03100
K03294
K02013
K03924
K07107
K00382
K00845
K02057
K07058
K00826
K01991
K02355
K02275
K01754
K00615
K10439
K03497
K03296
K03657
K09458
K08738
K04771
K01649
K04487
K03086
K00820
K05366
K02274
K01091
0.017
<0.001
<0.001
0.001
<0.001
0.004
0.010
<0.001
0.017
0.006
<0.001
<0.001
<0.001
<0.001
<0.001
0.001
<0.001
0.004
0.001
0.001
0.030
0.026
0.033
0.006
0.025
0.018
<0.001
0.020
0.029
0.034
<0.001
0.001
0.002
0.049
0.038
<0.001
<0.001
0.004
<0.001
<0.001
0.020
0.003
<0.001
<0.001
<0.001
<0.001
<0.001
0.001
0.037
<0.001
<0.001
<0.001
0.014
0.001
<0.001
<0.001
p_value
0
0.006
Means in groups
-0.001
-0.0005
0
0.0005
Difference between groups

Table S1: Sample details. The rhizosphere soil samples were collected from five grafted clonal varieties and from five trees of each variety.

Grafted clonal Spruce variety	Trees	L-site	P-site
1901	A	L1901_A	P1901_A
	B	L1901_B	P1901_B
	C	L1901_C	P1901_C
	D	L1901_D	P1901_D
	E	L1901_E	P1901_E
1902	A	L1902_A	P1902_A
	B	L1902_B	P1902_B
	C	L1902_C	P1902_C
	D	L1902_D	P1902_D
	E	L1902_E	P1902_E
1908	A	L1908_A	P1908_A
	B	L1908_B	P1908_B
	C	L1908_C	P1908_C
	D	L1908_D	P1908_D
	E	L1908_E	P1908_E
1941	A	L1941_A	P1941_A
	B	L1941_B	P1941_B
	C	L1941_C	P1941_C
	D	L1941_D	P1941_D
	E	L1941_E	P1941_E
1950	A	L1950_A	P1950_A
	B	L1950_B	P1950_B
	C	L1950_C	P1950_C
	D	L1950_D	P1950_D
	E	L1950_E	P1950_E

Table S2: Analysis of Similarity (ANOSIM) represents the variation in the bacterial and fungal communities in the rhizosphere soil samples between the two sites (L-site and P-site). The positive R values indicate significant differences in the microbial communities in the two soils. *P*-value < 0.05 represents significant differences.

Diversity	Group	R-value	P-value
Bacteria	L-site vs P-site	0.87533	0.001
Fungi	L-site vs P-site	0.37832	0.001

Excel S1 | Soil elements and Soil metabolites.

Excel S2 | Read counts_Raw and Clean reads

Excel S3 | Relative ASV table for bacterial and fungal communities

Excel S4 | Common and unique soil bacterial communities_Lipova_Prenet.

Excel S5 | Common and unique soil fungal communities_Lipova_Prenet.

Excel S6 | PICRUSt2_KO_Relative abundance

Excel S7 | FunGuild_Relative abundance of fungal guilds

Excel S8 | Bacterial co-occurrence network analysis_Nodes and Spearman Index

Excel S9 | Fungal co-occurrence network analysis_Nodes and Spearman Index
